# β-Carotene Attenuates Angiotensin II-Induced Aortic Aneurysm by Alleviating Macrophage Recruitment in *Apoe^−/−^* Mice

**DOI:** 10.1371/journal.pone.0067098

**Published:** 2013-06-27

**Authors:** Kaliappan Gopal, Perumal Nagarajan, Jose Jedy, Avinash T. Raj, S. Kalai Gnanaselvi, Parveen Jahan, Yogendra Sharma, Esaki M. Shankar, Jerald M. Kumar

**Affiliations:** 1 Center for Cellular and Molecular Biology, Hyderabad, India; 2 National Institute of Immunology, New Delhi, India; 3 Department of Genetics, Osmania University, Hyderabad, India; 4 Department of Orthopedics, National Orthopaedics Center for Excellence in Research and Learning, Faculty of Medicine, University of Malaya, Kuala Lumpur, Malaysia; 5 Tropical Infectious Disease Research and Education Center, Department of Medical Microbiology, Faculty of Medicine, University of Malaya, Kuala Lumpur, Malaysia; Max-Delbrück Center for Molecular Medicine (MDC), Germany

## Abstract

Abdominal aortic aneurysm (AAA) is a common chronic degenerative disease characterized by progressive aortic dilation and rupture. The mechanisms underlying the role of α-tocopherol and β-carotene on AAA have not been comprehensively assessed. We investigated if α-tocopherol and β-carotene supplementation could attenuate AAA, and studied the underlying mechanisms utilized by the antioxidants to alleviate AAA. Four-months-old *Apoe^−/−^* mice were used in the induction of aneurysm by infusion of angiotensin II (Ang II), and were orally administered with α-tocopherol and β-carotene enriched diet for 60 days. Significant increase of LDL, cholesterol, triglycerides and circulating inflammatory cells was observed in the Ang II-treated animals, and gene expression studies showed that *ICAM-1, VCAM-1, MCP-1, M-CSF, MMP-2, MMP-9* and *MMP-12* were upregulated in the aorta of aneurysm-induced mice. Extensive plaques, aneurysm and diffusion of inflammatory cells into the tunica intima were also noticed. The size of aorta was significantly (P = 0.0002) increased (2.24±0.20 mm) in the aneurysm-induced animals as compared to control mice (1.17±0.06 mm). Interestingly, β-carotene dramatically controlled the diffusion of macrophages into the aortic tunica intima, and circulation. It also dissolved the formation of atheromatous plaque. Further, β-carotene significantly decreased the aortic diameter (1.33±0.12 mm) in the aneurysm-induced mice (β-carotene, P = 0.0002). It also downregulated *ICAM-1, VCAM-1, MCP-1, M-CSF, MMP-2, MMP-9, MMP-12, PPAR-α* and *PPAR-γ* following treatment. Hence, dietary supplementation of β-carotene may have a protective function against Ang II-induced AAA by ameliorating macrophage recruitment in *Apoe^−/−^* mice.

## Introduction

Abdominal aortic aneurysm (AAA) is a common chronic degenerative disease involving the aortic wall in human. It is estimated that approximately 10% of elderly men show localized structural deterioration of the aortic wall, leading to progressive aortic dilation and rupture [1], [2]. Accumulating body of evidence indicate that AAA is influenced by various risk factors including family history, smoking, aging, lifestyle and hypertension [1], [3]. Nonetheless, it is also increasingly becoming clear that certain hematological factors also significantly influence the development of AAA [3]. Of note, angiotensin II (Ang II) is one of the key regulatory peptide implicated in the pathogenesis of certain cardiovascular diseases, particularly AAA [4]. Recent lines of evidence suggest that Ang II could trigger intracellular accumulation of reactive oxygen species (ROS) culminating in the initiation of lipid peroxidation-mediated oxidative stress [5]. ROS is involved in myriad of downstream signaling pathways viz., transcription factors, tyrosine kinases, protein kinases, ion channels and certain other potential molecular targets [6]. ROS reportedly transduce cell growth, apoptosis and cell migration, and affect expression of inflammatory and extracellular matrix (ECM) genes [6]. Furthermore, substantial experimental evidence suggests that ROS could induce the expression of certain matrix metalloproteinases (MMPs) [7], plasma soluble adhesion molecules e.g. intercellular adhesion molecule-1 (ICAM-1) and vascular cell adhesion molecule-1 (VCAM-1), activation of chemokines, e.g. macrophage chemotactic protein-1 (MCP-1) and macrophage-colony stimulating factor (M-CSF), contributing to the onset of vascular inflammation [2], [3]. Changes in the normal vascular endothelium of older individuals and in atherosclerotic lesions are believed to be due to the onset of oxidative stress and increase of ROS [7]. Although it is sufficiently evident that lipid peroxidation and transcriptional factors play a major role in determining the onset of atherosclerosis, potential therapeutic targets against AAA still remain controversial. Several therapeutic strategies tested in large clinical trials conducted in the past have failed to conclusively improve disease prognosis, and currently no effective treatment modules are available against AAA [8], [9]. Although open surgical repair can markedly improve disease prognosis in patients with large AAAs, invasive surgical procedures and associated complications are critical limiting factors, especially in old age [1].

Antioxidants are widely used in the prevention of diseases associated with oxidative stress and atherosclerosis [10], [11]. Among the antioxidants, α-tocopherol and β-carotene have been widely investigated in experimental atherosclerosis in *Apoe^−/−^* mice, and are believed to have strong free radical-scavenging properties [5]. However, reports on their abilities to protect against LDL-mediated peroxidation remain elusive. Studies have established that pre-treatment of β-carotene inhibits atherosclerosis [12]. However, others suggest that although β-carotene could alleviate atherosclerotic lesions, it has no effect on LDL oxidation [11], [13], [14]. α-tocopherol is an essential fat-soluble micronutrient in higher mammals. Apart from its established role as an antioxidant for lipids, it also functions as a key regulator of gene expression, cell signaling and proliferation [15]. However, the synergistic effect of antioxidants viz., α-tocopherol and β-carotene on inflammatory cells, gene expression, especially adhesion molecules, plasminogen activator and inhibitor system, MMPs, metabolic and molecular changes in different organ systems remain elusive. Further, the mechanisms underlying the functions of α-tocopherol and β-carotene in AAA have not been comprehensively assessed. Here, we investigated if independent and combined antioxidant supplementations of α-tocopherol and β-carotene could attenuate AAA. We also determined if these dietary antioxidants could impact the recruitment of inflammatory cells including macrophage, lymphocytes, and serum total cholesterol and LDL levels in the circulation. Furthermore, we also evaluated the molecular factors utilized by these antioxidants to control AAA in Ang II-induced as well as antioxidant-treated *Apoe^−/−^* mice, and the specific receptors employed by α-tocopherol and β-carotene to control AAA disease progression.

## Results

### Angiotensin II Treatment Induces Abdominal Aortic Aneurysm in *Apoe^−/−^* Mice

**Figure 1:**
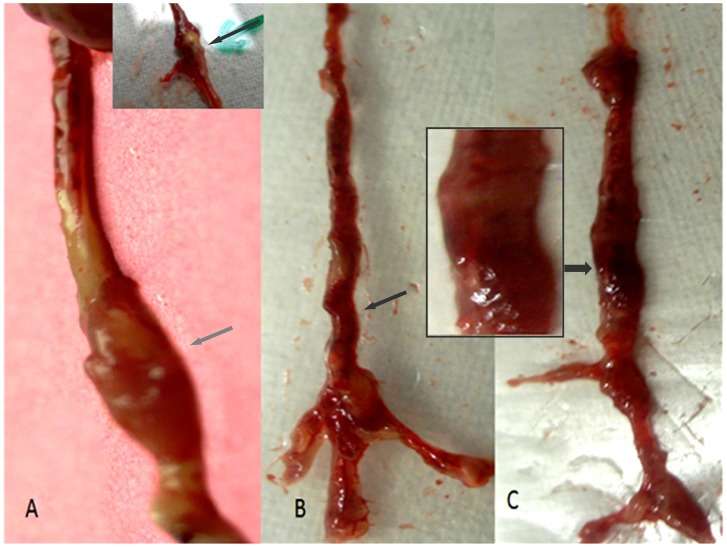
Induction of abdominal aneurysm in *Apoe−/−* mice. (**A**) Abdominal aneurysm observed in the Ang II-treated *Apoe−/−* mice; (**B**) Plaque reduction in the entire aorta and pseudoaneurysm observed in the abdominal and thoracic aorta of β-carotene- treated animals; (**C**) Abdominal aneurysm resembles with Ang II-treated mice but plaque formation was reduced in the α-tocopherol-treated animals.

Ang II-infused *Apoe^−/−^* mice showed balloon-like dilation and extensive plaque formation in the thoracic and abdominal aorta, whereas control *Apoe^−/−^* mice did not develop any signs of AAA [16]. Aneurysm was mostly seen in the suprarenal portion of abdominal aorta, and plaque extension was predominantly seen in the abdominal and thoracic aorta. Our recent findings showed that no lesions were seen in the aortic arch, branching of the subclavian, renal and femoral arteries [17]. Here, we observed amelioration of plaque in entire aorta and onset of pseudo-aneurysm in the abdominal and thoracic aorta of β-carotene-treated *Apoe^−/−^* mice (**Fig. 1A, B**). α-tocopherol treated animals showed aneurysm resembling those of Ang II-treated *Apoe^−/−^* mice, although the extent of plaque formation was minimal (**Fig. 1C**). Details of size of abdominal aorta diameter have been shown in **Table 1**. The size of aorta was significantly (P = 0.0002) increased (2.24±0.20 mm) in the aneurysm-induced animals as compared to control mice (1.17±0.06 mm). Further, β-carotene significantly decreased the aortic diameter (1.33±0.12 mm) in the aneurysm-induced *Apoe^−/−^* mice (β-carotene, P = 0.0002).

**Table 1:** The differences in size of aortic lesions/aortic wall between the treated and untreated *Apoe^−/−^* mice (represented in mm).

Treatment	Aortic Diameter (mm)	P value†	
Control	1.17±0.06	Angiotensin II vs Control	0.0002
Angiotensin II	2.24±0.20		
α-tocopherol	1.72±0.09	Treatment vs Angiotensin II	0.0007
β-carotene	1.33±0.12		0.0002
α-tocopherol+β-carotene	1.473±.05		0.0003

† P values of <0.05 were considered significant.

### Supplementation of β-carotene and α-tocopherol does not Reduce LDL Levels in Aneurysm-induced *Apoe^−/−^* Mice

**Figure 2:**
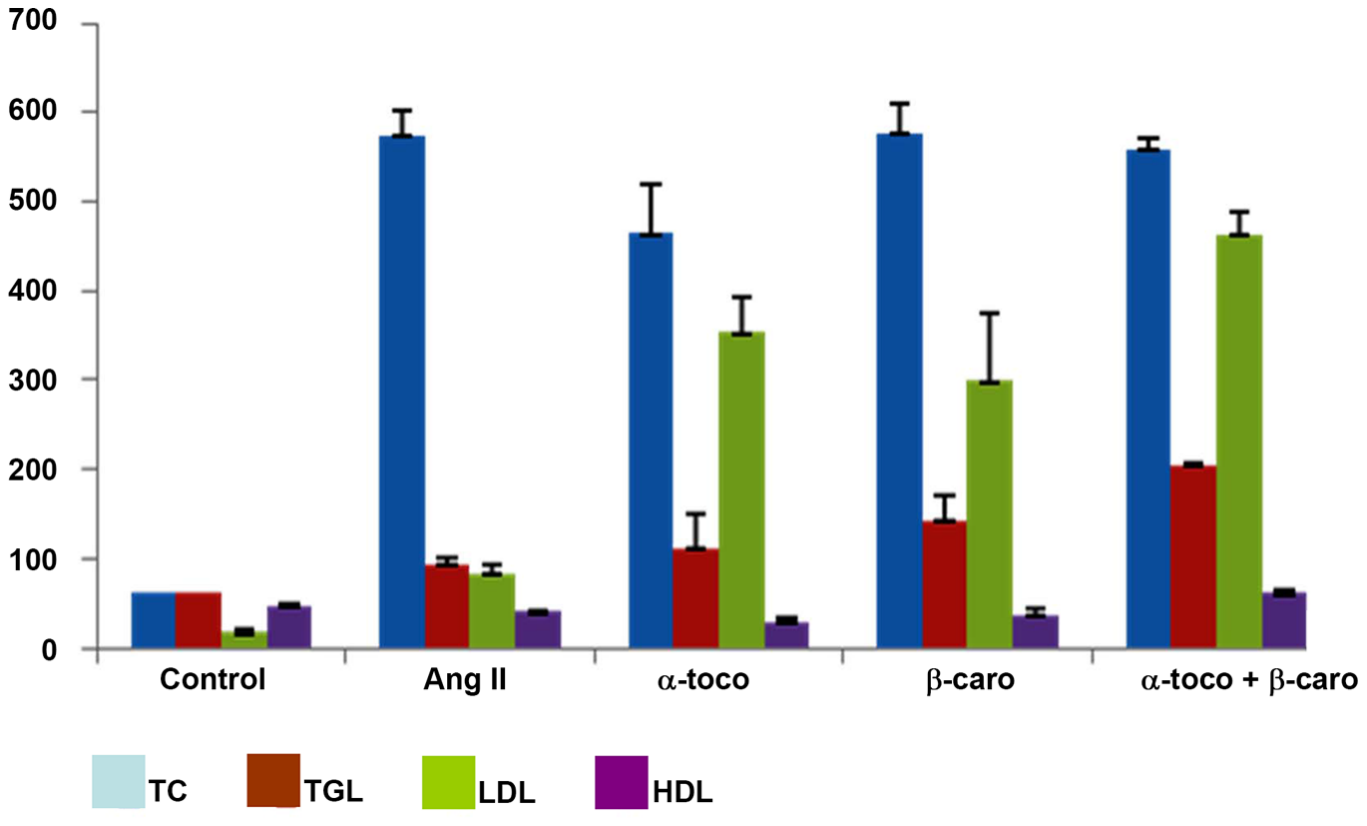
Biochemical analysis of the *Apoe−/−* experimental groups. Serum TC, triglycerides and LDL levels were analysed in the different study groups. (Ang II-treated (n = 6), β-carotene-treated (n = 6) and control (n = 6). P values of less than 0.05 were considered significant.

It is widely believed that β-carotene and α-tocopherol function as efficient antioxidants. However, their potential role in alleviating the levels of LDL still remains controversial in experimental aneurysm. Hence, we set out to estimate the LDL levels in serum of the experimental animals under investigation. Detailed biochemical analysis of Ang II-treated, β-carotene-treated and control *Apoe^−/−^* mice is shown in **Fig. 2**. We found that serum TC, triglycerides and LDL levels were significantly increased (P<0.05) in Ang II-treated animals when compared with saline-treated *Apoe^−/−^* mice. However, the levels of HDL were decreased, but not significantly in Ang II-treated *Apoe^−/−^* mice. Significant increase in the levels of LDL, triglycerides and decrease in the levels of HDL was observed in α-tocopherol-treated animals when compared with Ang II-treated *Apoe^−/−^* mice. Significant increase (P<0.05) in LDL levels, increase cholesterol, and triglyceride levels and decrease in HDL levels were evident in β-carotene-treated animals as compared with Ang II-treated *Apoe^−/−^* animals. Further, significant increase of LDL, HDL, triglycerides and increase of cholesterol levels was observed in the combined antioxidant-treated mice relative to the Ang II-treated animals. The lipid profile of the different study groups is shown in **File S1**. Based on the above observations, α-tocopherol, β-carotene and combined antioxidant treatments did not reduce the lipid profiles in *Apoe^−/−^* mice [18], but two to three-fold increases in the levels of LDL, and triglycerides were observed in the treated *Apoe^−/−^* mice as compared with the control mice.

### β-carotene Treatment Resolves Atheromatous Plaques and Macrophage Infiltration into the Aorta of AAA-induced *Apoe^−/−^* Mice

**Figure 3:**
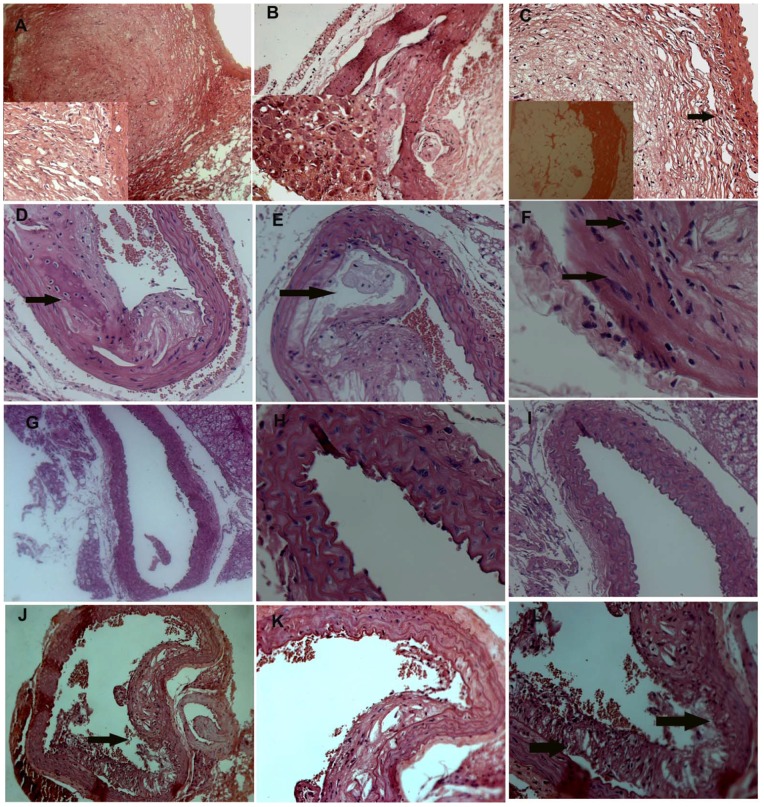
Histopathological analysis of abdominal aortic tissues. (**A–C**)**Ang II treatment.** Multiple foci of adhesive atheroma formation seen in the tunica intima. Severe vacuolar and fatty degeneration and accumulation, and invasion of modified macrophages (foam cells) are also seen; (**D–F**) **β-carotene treatment.** Mild to moderate dissolution of atheromatous plaque in the tunica intima. Foci of calcified region (box) in which sheet like structure composed of chondrocytelike cells; collagen and inflammatory cells in the intimal layer of aorta (arrow) are seen; (**G–I**) **α-tocopherol treatment.** Complete atheromatous plaque dissolution is visible in the intimal part of aorta. (**J–L**) **Combined antioxidant treatment.** Mild disintegration or dissolution of atheromatous plaques and thrombus (arrow) are seen.

Inflammatory cell infiltration is reported to be the hallmark of aortic aneurysm. The role of antioxidants in containing inflammation in aortic vessels has seldom been investigated. Therefore, we determined the credentials of β-carotene in controlling the recruitment of inflammatory cell infiltration in AAA. In gross pathology, extensive plaque reduction was noticed along the entire aorta and pseudoaneurysm was noticed in the abdominal and thoracic aorta of β-carotene-treated as well as of combined antioxidant-treated *Apoe^−/−^* mice. However, we did not find any prominent signs of aneurysm in the entire aorta of the β-carotene-treated as well as combined antioxidant-treated mice when compared with Ang II-treated *Apoe^−/−^* mice (**Fig. 3A–C**). These gross observations suggest that β-carotene treatment could dissolve the atheromatous plaques in the aneurysm-induced *Apoe^−/−^* mice. These observations were further confirmed by detailed histopathological investigations, where we found that the use of β-carotene completely resolved the zones of atheromatous plaque in the aorta and disruption of endothelial layer was evident in the aortic tunica intima (**Fig. 3D–F**).

Infiltration of macrophage and lymphocytes play a crucial role in inflammation. Notably, infiltration of inflammatory cells was relatively lesser in the aortic tunica intima of β-carotene-treated *Apoe^−/−^* mice. Ang II-treated animals showed severe degenerative and inflammatory changes in thoracic and abdominal aorta. Multiple foci of adhesive atheroma formation were noticed in the intimal layer. The predominant degenerative changes were vacuolar and fatty changes seen in the tunica intima of abdominal aorta. Interestingly, the tunica intima of abdominal and thoracic aorta was entirely replaced with macrophages. Moreover, the cytoplasm of these cells had enormous accumulation of amorphous fatty exudates resembling foam cells in the Ang II-treated *Apoe^−/−^* mice. We did not find any atheromatous plaque replacement in the α-tocopherol-treated group. However, mild to moderate dissolution of atheromatous plaques in the endothelial layer of intimal region of aorta was seen. Further, numerous inflammatory cells were seen to invade the atheromatous plaque (**Fig. 3G–I**). In addition, considerable level of inflammatory cells and calcification were also evident in the tunica media of aorta in the α-tocopherol-treated animals compared with Ang II-treated *Apoe^−/−^*mice. Along the foci of calcified region, chondrocyte-like cells surrounded by collagen and inflammatory cells were observed in the intimal and medial layers of aorta (**Fig. 3G–I**). These observations strongly imply that α-tocopherol treatment did not replace the atheromatous plaque in the aorta of Ang II-treated *Apoe^−/−^* mice. However, combined antioxidant treatment resulted in mild disintegration or dissolution of atheromatous plaques and thrombus and invasion of lesser number of inflammatory cells into the aortic tunica media (**Fig. 3J–L**).

### Supplementation of β-carotene Leads to Decreased Levels of Circulating Macrophages and Lymphocytes in AAA-induced *Apoe^−/−^* Mice

**Figure 4:**
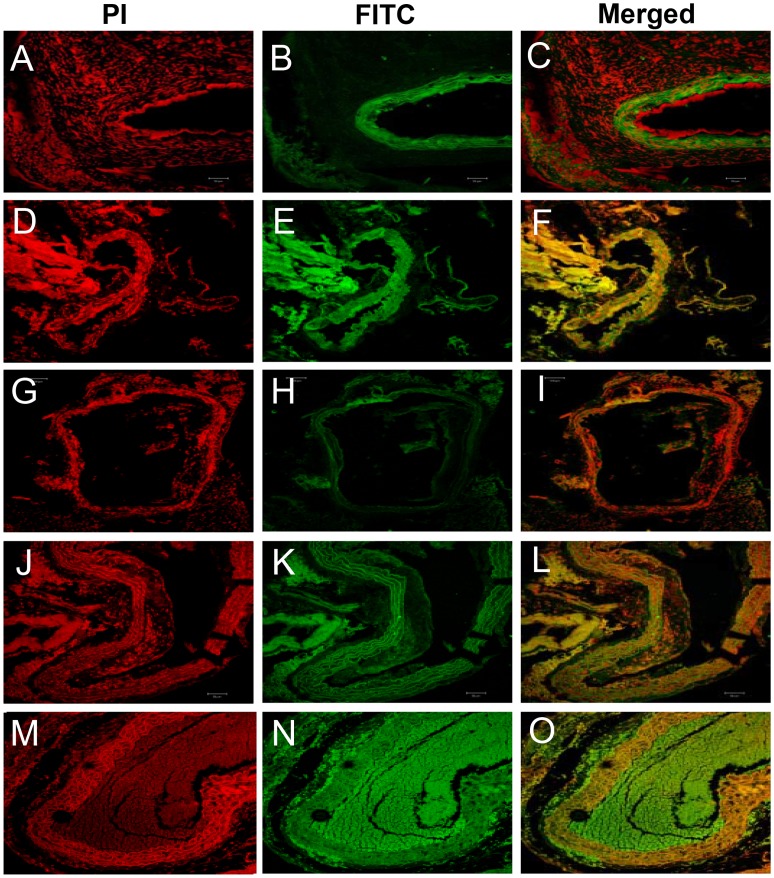
Confocal microscopy of CD45.2 expression in aortic tissue sections of *Apoe−/−* mice. (**A–C** CD45.2 Ang II- treated group) 45.2 over-expression of CD45.2 protein seen in the atheromatous plaques in the tunica media of Ang II- treated Apoe−/− mice; (**D–F** CD45.2 α-tocopherol-treated**)** Mild levels of expression are seen in α-tocopherol-treated group; (**G–I** CD45.2 β-carotene-treated**)** CD45.2 is not expressed in the β-carotene-treated group; (**J–L CD45**.2 α-tocopherol+β-carotene-treated) Combined treatment shows mild expressions of CD45.2 (**M–O** Control).

Next, we sought to determine if antioxidant treatment could impact the levels of circulating inflammatory cells in AAA. We found that α-tocopherol significantly decreased (P<0.05) the levels of Mac3 levels as compared with control *Apoe^−/−^* mice (**Table 2**). Further, the levels of macrophages were significantly decreased (*P = 0.03*) in the β-carotene-treated and combined antioxidant-treated animals relative to control *Apoe^−/−^* mice. This observation suggests that both antioxidants could play a synergistic role in regulating circulating macrophages in AAA. We also found that β-carotene significantly restored circulating lymphocytes to normal levels in Ang II-treated *Apoe^−/−^* mice. In contrast, α-tocopherol and combined antioxidant treatment further increased the lymphocyte levels in Ang II-infused *Apoe^−/−^* mice (**Table 2**). This vindicates that β-carotene effectively control the level of circulating inflammatory cells in AAA (**Fig. 4** **and** **5**).

**Figure 5:**
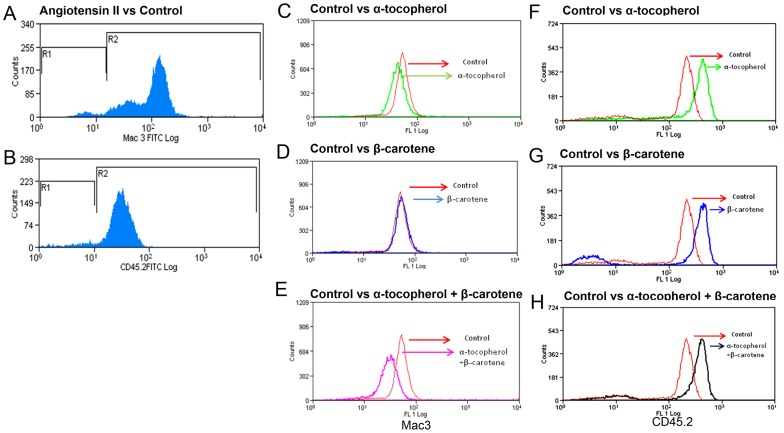
(A) FACS analysis of peripheral blood against Mac3. Significant increase in the Mac3 positive cells (R2) in Ang II-treated animals compared with control (R1); (**B**) **FACS analysis of peripheral blood against CD45.2.** Slight increase in the percentage of CD45.2 cells is seen in the Ang II-treated (R1) animals compared with control (R1); (**C–E**) **FACS analysis of peripheral blood against Mac3.** α-tocopherol-treatment slightly decreased Mac3 expression whereas no marked difference is seen in the β-carotene-treated group. Combined treatment shows downregulation of Mac3 on macrophages; (**F–H**) **FACS analysis of peripheral blood against CD45.2.** No marked difference is seen between the control and antioxidant-treated groups with CD45.2 expression.

**Table 2:** FACS data of blood inflammatory macrophages and lymphocytes from β-carotene and α-tocopherol-treated mice.

Experimental Group	CD45.2	Mac3
**β-carotene**	78±1.24	91.65±0.495
**α-tocopherol**	83.15±0.78	90.75±0.354
**β-carotene+α-tocopherol**	84.3±2.40	84.3±2.40
**Control**	78±0.71	93.4±0.283

### Combined Antioxidant-treatment Downregulates Expression of Mac3 and CD45.2 in Resident Macrophages of AAA-induced *Apoe^−/−^* Mice

**Figure 6:**
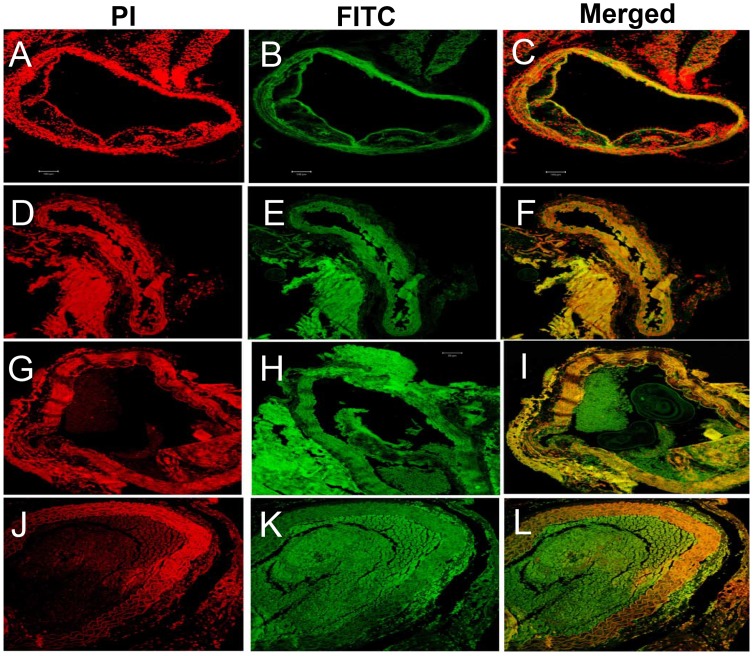
Confocal microscopic images of expression of Mac3 in the aorta of *Apoe−/−* mice in the different treatment groups. (**A–C** Mac3- Ang II-treated) Mac3 is over-expressed in the atheromatous plaques of aortic tunica media of Ang II-treated mice; (**D–F** Mac3-α-tocopheroltreated & G- I Mac3- β-carotene- treated) Dissoluted or loosely cohesive plaques seen in the tunica media in the antioxidant-treated group; (**J–L** Control).

To further investigate the effect antioxidant treatment has on Mac3 and CD45.2 in macrophages, we evaluated the protein expression levels in the aorta of Ang II and antioxidant-treated *Apoe* ***^−/−^*** mice. Mac3 and CD45.2 proteins were upregulated in the monocyte/macrophages and lymphocytes of the aortic tunica intima of the Ang II-treated *Apoe^−/−^* mice. We also found an increase in the number of macrophages and lymphocytes infiltrating into the lamella of tunica media that also expressed high levels of Mac3 (**Fig. 6A–C**) and CD45.2 (**Fig. 4A–C**). We also found that the loosely cohesive plaques and dissolved atheromatous plaques in the tunica intima of β-carotene-treated *Apoe^−/−^* mice showed mild expressions of CD45.2 and Mac3 in the foam cells and lymphocytes surrounded by collagen. Of note, a major portion of atheromatous plaque was dissolved where the intimal region appeared normal in β-carotene-treated *Apoe^−/−^* mice (**Fig. 4G–I** **and** **6G–I**). The above observations suggest that β-carotene may have crucial role on plaque dissolution and prevention of atheroma formation. However, two important inflammatory markers CD45.2 and Mac3 were upregulated in the inflammatory foci of α-tocopherol-treated abdominal aorta. Inflammatory foci displayed microplaques adherent to medial layer, surrounded by elastic lamella infiltrated with foam cells and lymphocytes (**Fig. 4D–F** **and** **6D–F**). It was also clearly established that the β-carotene-treated *Apoe^−/−^* mice did not express CD45.2, whereas the combined treatment group showed mild levels of expression (**Fig. 4J–L**).

**Figure 7:**
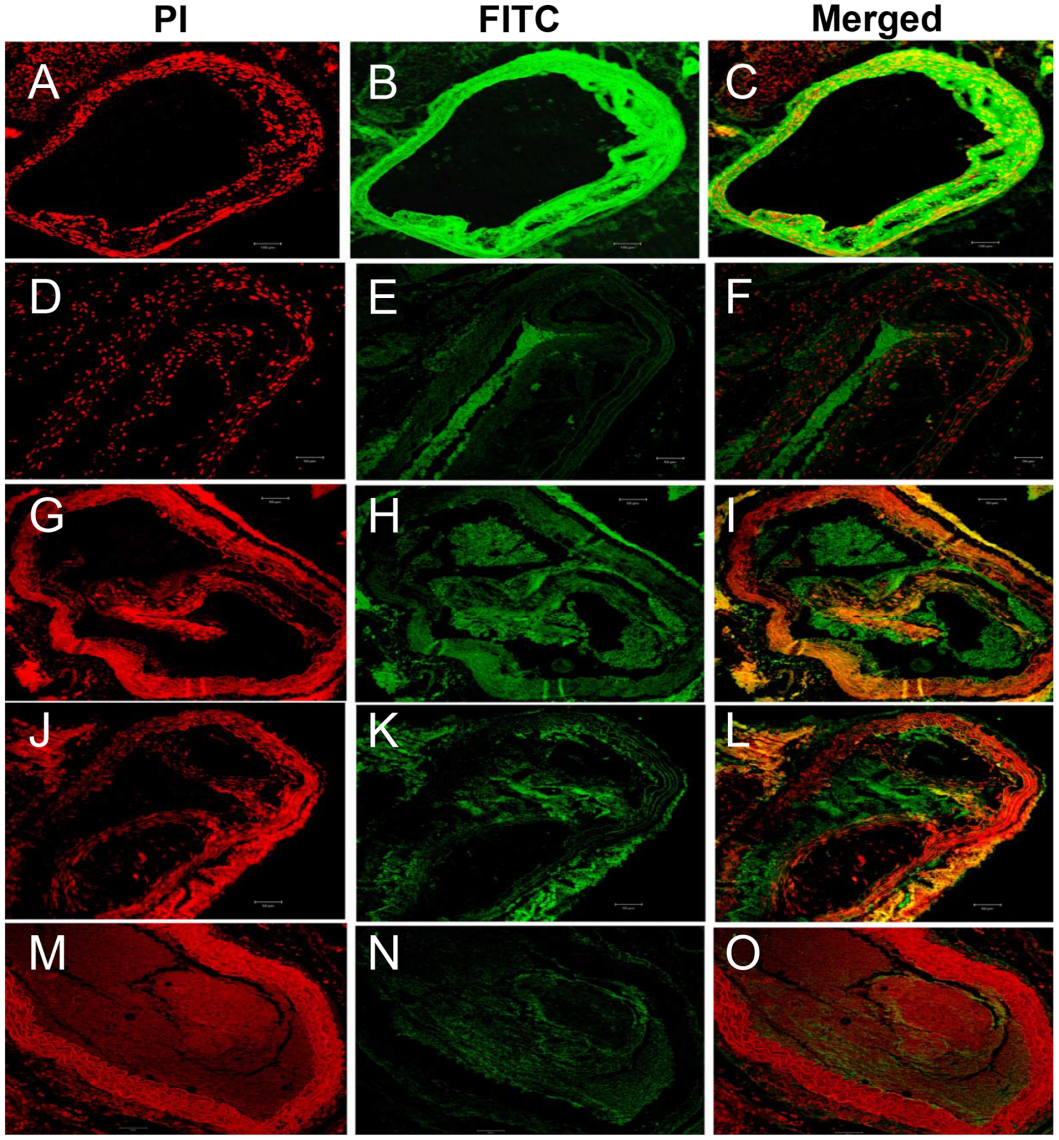
Confocal microscopic images of expression of ICAM-1 (CD54) in the aorta of *Apoe−/−* mice. (**A–C** ICAM-1 Ang II-treated) Over-expression of ICAM-1 protein is seen in the atheromatous plaques of tunica intima of Ang II-treated animals. (**D–F** ICAM-1 α -tocopheroltreated) ICAM-1 expression is not seen in the α-tocopherol- treated group; (**G–L** ICAM-1 β-carotene-treated) Less expression of ICAM-1 is seen in the dissoluted or loosely cohesive plaques of tunica intima of both β-carotene-treated and combined antioxidant-treated animals. (**MO**) Control.

Our further investigations on VEGF and ICAM-1 proteins showed that the proteins were upregulated in the atheroma of aortic tunica intima and media, and the endothelial and smooth myocytes of Ang II-treated animals as compared with control *Apoe^−/−^* mice (**Fig. 7A–C** **and** **8A–C**). The expression of VEGF and ICAM-1 proteins was relatively lesser in *Apoe^−/−^* mice treated with antioxidants, particularly β-carotene as compared to Ang II-treated animals (**Fig. 7D–F** **and** **8G–L**). Furthermore, we also observed upregulated RAR-α in the aorta of β-carotene or combined antioxidant-treated *Apoe^−/−^* mice relative to Ang II-treated and α-tocopherol-treated animals (**Fig. 9**).

**Figure 8:**
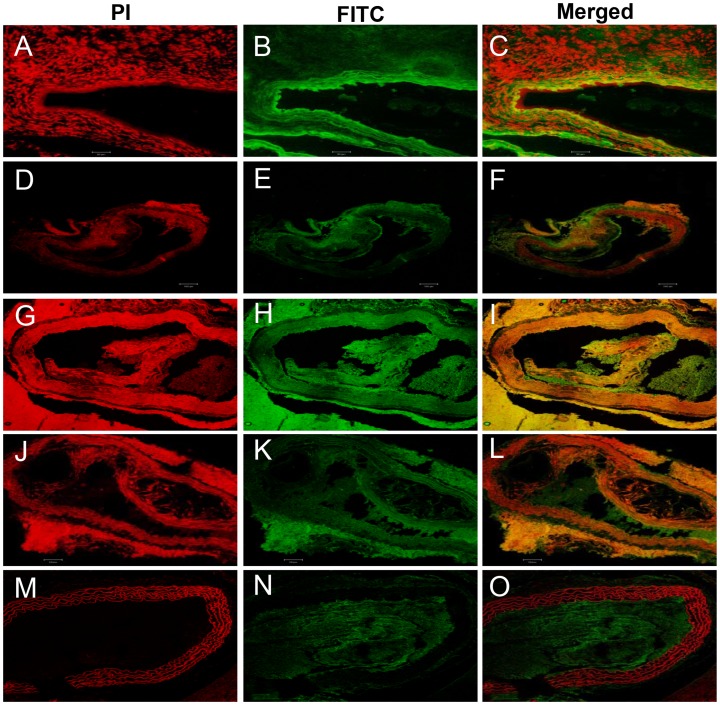
Confocal microscopic images of expression of VEGF in the aorta of *Apoe−/−* mice. (**A–C** VEGF Ang II-treated) Upregulation of VEGF protein is seen in the atheromatous plaques of tunica intima of Ang II-treated animals. (**D–F** VEGF α-tocopherol-treated) VEGF expression is not seen in the α-tocopherol-treated group. (**G–L**) Lesser expression of VEGF is seen in the dissolved or loosely cohesive plaques of tunica intima of both β-carotene-treated and combined antioxidant-treated animals. (**M–O**) Control.

**Figure 9:**
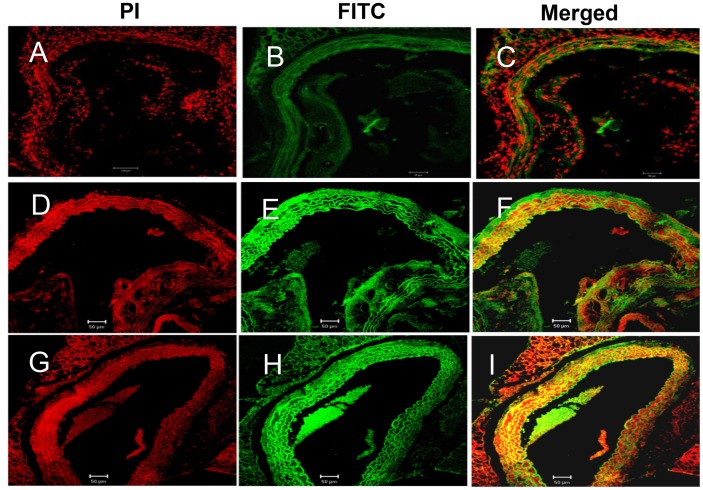
Confocal microscopic images of expression of RAR in the aorta of *Apoe−/−* mice. (**A–C** RAR Ang II-treated) RAR expression is not seen in the Ang II-treated group; (**D–I**) Over-expression of RAR- α is observed in the dissoluted and loosely cohesive atheromatous plaques of tunica intima of both groups treated with α-tocopherol and β-carotene antioxidants.

### Angiotensin II Induces Aneurysm by the Upregulation of MMP, PPAR-γ, MCP-1 and uPAR mRNA Expression

Given the role of adhesion molecules in the pathogenesis of atherosclerosis, we investigated the effect of *ICAM-1* and *VCAM-1* in our present analysis. Herein, we found that the mRNA expression of *ICAM-1* and *VCAM-1* were upregulated to 1.3 and 4.4-fold respectively in Ang II-treated aorta of *Apoe^−/−^* mice. The expression pattern of MMP family members indicated that *MMP-2, MMP-9* and *MMP-12 *were upregulated 2, 14 and 17-folds respectively. Amongst the members of *PAS, the uPA* receptor was dramatically upregulated to 51-fold, and *PPAR-γ* to 32-fold. Of note, *MCP-1, M-CSF* and *Ren1* were upregulated in Ang II-treated *Apoe^−/−^* mice. Hence, it is clear that Ang II treatment resulted in the upregulation of *MMP, PPAR-γ, MCP-1* and *uPAR* messages in experimental AAA. The pattern of mRNA expression of the different candidate genes is shown in **Table 3**.

**Table 3:** mRNA expression profile of different candidate genes in angiotensin II-treated and antioxidant-treated *Apoe−/−* mice (given in fold expression).

Gene/molecule		Fold Expression in Different Treated Groups		
		Ang II	α-tocopherol	β-carotene
**Adhesion molecules**	*ICAM-1*	1.3	26.54	19.84*
	*VCAM-1*	4.4	53.45	6.59
**Chemokines/cytokines**	*MCP-1*	4.6	1.54	3.25*
	*M-CSF*	3.2*	206.5	17.75
**Matrix metalloproteinases**	*MMP-2*	1.9	57.28	3.63
	*MMP-9*	13.91	1.44*	7.57*
	*MMP-12*	113	1.51*	2.89
**Plasminogen activation system**	*tPA*	1.7*	4.79*	11.63*
	*uPA*	1.5	17.15*	10.63*
	*PAI*	1.6	576.03	23.75
	*uPAR*	51	3.76	8.46*
**Peroxisome-proliferator activated receptors**	*PPAR-α*	1.7*	4.44	1.02*
	*PPAR-G*	32	604.67*	27.28*
**Retinoic acid receptors**	*RAR-α*	3.45	13.92*	16.67
	*RAR-β*	3.58	5.24*	13.73
	*RAR-γ1*	93.05	7.72	39.12
	*RAR-γ2*	27.66	5.65	8
**Retinoid X receptor**	*RXR-α*	5.81	5.89*	39.12
	*RXR-β*	113.77	5.85	118.62
	*RXR-γ*	219.79	83.86	12.38
	*Ran1*	5	15.45	1.43*

* Indicates mRNA downregulation.

### β-carotene Represses the Expression of Certain Inflammatory Genes in AAA-induced *Apoe^−/−^* Mice

Next, we studied the mRNA levels of *ICAM-1* and *VCAM-1* and found that their levels were increased in the aorta of α-tocopherol-treated animals relative to Ang II-treated animals. Interestingly, adhesion molecules, cytokines, PAS and peroxisome proliferator-activated receptors (PPARs) were markedly increased in α-tocopherol treated as compared to Ang II-treated animals. However, *MMP-2, MMP-9, MMP-12* and *uPAR* mRNA levels were significantly decreased following α-tocopherol treatment. Overall, the results suggest that α-tocopherol did not play a salubrious role in regulating the key genes associated with AAA. We observed that combined antioxidant use downregulated *MMP-2, MMP-9* and *MMP-12* in the aorta relative to Ang II-treated animals. We also found that the chemokines *MCP-1* and *M-CSF* were downregulated in β-carotene treated relative to Ang II-treated animals. Further, we also observed downregulation of *Ren1* message. This observation is indicative that β-carotene facilitates the regeneration of tubular region of kidney in AAA. Interestingly, β-carotene downregulated the PASs messages (*uPA, tPA* and *uPAR*), and upregulated the PAI-1 systems. This observation indicates that β-carotene could function via a pathway independent of the plasminogen system to dissociate plaque/atheroma formation. The pattern of mRNA expression of the different candidate genes is shown in **Table 3**.

## Discussion

The mouse Ang II infusion model of AAA formation is reportedly the best model for its prospective relevance to human AAA [15], [18]. Herein, we have convincingly demonstrated that dietary supplementation of α-tocopherol and β-carotene resulted in substantial protection of Apoe−/− mice from AAA. Similar to AAA in human, AAA in experimental animals used herein, also showed high cholesterol, LDL, inflammatory cell levels and histological features, for instance leukocyte infiltration and plaque formation [1]. Several studies have established that α-tocopherol and β-carotene confers protection from LDL-mediated oxidative stress in vitro [19], [20]. On the contrary, others have suggested that dietary supplementation of α-tocopherol and β-carotene has no effect on LDL oxidation in atherosclerosis-induced mice. However, their effect on AAA still remains indefinite [12], [13]. Herein, we have convincingly shown that α-tocopherol significantly reduced (P<0.05) the levels of the circulating macrophages in the blood. It is also clear that the number of macrophages infiltrating into the tunica media were significantly decreased in the α-tocopherol-treated animals as compared to Ang II-treated animals. Although, the histopathological features are suggestive of moderate dissolution of atheromatous plaque in the aortic tunica intima, invasion of large number of inflammatory cells into the atheromatous plaque and calcification is suggestive of the inability of independent dietary α-tocopherol in controlling AAA. Further, the significant decrease of cholesterol levels noticed in α-tocopherol-treated mice although not to beneficial levels as compared with Ang II group, has previously been shown in rats and rabbits [21], but not in human [22].

This investigation describes the salubrious role of β-carotene on Ang II-induced AAA in the *Apoe^−/−^*mouse models. We found that β-carotene supplementation, markedly controls the level of circulating macrophages and lymphocytes compared with Ang II-induced animals. We also observed complete resolution of atheromatous plaque in the aorta of β-carotene-treated mice, although limited number of invading inflammatory cells was also seen in these regions. Gross pathological investigations divulged extensive plaque reduction in the entire aorta and pseudoaneurysm in the abdominal and thoracic aorta in animals treated with β-carotene or combined α-tocopherol and β-carotene. However, serum LDL levels were increased in the antioxidant-treated group, a similar observation also documented previously by others [12], [13]. This is indicative that the effect of β-carotene on AAA is not influenced by LDL oxidation. Further, it is also evident that β-carotene might influence AAA by controlling the magnitude of inflammation, improving the immune system and conferring beneficial functions on transcriptional factors [3]. In the current analysis, we have found that β-carotene acted by decreasing circulatory inflammatory cells both in AAA, and from the circulation as evident from histopathological investigations, FACS and immunofluorescence experiments. This is in agreement with earlier reports having shown that macrophage recruitment could be markedly controlled by β-carotene [23].

To the best of our understanding, large gene profiling analyses on the role of antioxidants in AAA have seldom been reported earlier. A limited number of aortic samples of patients with AAA post-antioxidant treatment have been analyzed for gene expression [1]. In the present investigation, both pre- and post-treatment gene expression profiling have yielded some basic insights into use of α-tocopherol and β-carotene against macrophage-related destructive factors *MCP-1, MMP-2, MMP-9, MMP-12 *in AAA. We observed increased expression of *VCAM-1, MCP-1, MMP-2, MMP-9, MMP-12, PPAR-γ* and *uPAR* in AAA. Our present data already suggest that these inflammatory genes may be highly relevant in the pathogenesis of atherosclerosis. ECM degradation is believed to play a vital role in aortic wall attenuation and subsequent onset of aneurysm. Several studies have established that Ang II-induced ROS generation is likely to trigger the expression of *MMP*s, including *MMP-2, MMP-9* and *MMP-12 *that reportedly play a central role in the degradation of various ECM proteins associated with AAA [24]–[26]. Investigations conducted in transgenic rodent models have confirmed the functional significance of *MMP-2, MMP-9* and *MMP-12 *in the onset of AAA. An important issue here is the clinical relevance of our findings, because increased *MMP-9* has also been demonstrated in tissues of human AAAs [21], [27]. In line with previous findings, we have found that *MMP-2, MMP-9* and *MMP-12* were increasingly upregulated in Ang II-treated AAAs. Of note, it was also seen that the mRNA expressions of *MMP-2, MMP-9* and *MMP-12* in AAA were markedly decreased in animals treated with α-tocopherol and β-carotene either individually or in combination.

PPARs are nuclear receptors that regulate lipid and glucose metabolism and cellular differentiation, activated by fibrates, fatty acids, and eicosanoids. Experimental evidence suggest that both, *PPAR-γ* and *PPAR-α* are expressed in human macrophages where they exert anti-inflammatory effects [28], [29]. In this regard, it is noteworthy that recent studies suggested that PPAR-α deficiency could lead to suppression of inflammation [28]–[30]. Furthermore, studies have confirmed that these nuclear receptors may also facilitate development of atherosclerosis [28]. In the current investigation, we have found upregulation of *PPAR-α* and *PPAR-γ* in AAAs, which however, were downregulated in animals treated with β-carotene alone or in combination with α-tocopherol suggesting the role of β-carotene in downplaying *PPAR* functions.

All PPAR isomers are involved in gene expression in multiple aspects of fat metabolism [31]. PPARs heterodimers with RXR and are required for high-affinity binding and recognition of PPAR elements to activate or repress target gene transcription. RA does not activate *PPAR-α* and *PPAR-γ* but, apocarotenals repress *PPAR* and *RXR* activation [30], [32]. PPARs inhibit the expression of myriad of genes in adipose tissue (*MMP-9, MMP-2* and *leptin*) and various transcription factors such as *NFκb* leading to antiinflammatory effects [33]. Similarly, our data also showed that β-carotene activated *RXRs* and *RARs* in the aorta of *Apoe^−/−^* mice and it may bind to PPARs to activate or repress the target genes through *PPARE*. Ang II-induced ROS is a potential regulator of *ICAM-1* and *VCAM-1* that are upregulated following NADPH oxidase activation. It is also to be recalled that activation of these adhesion molecules contribute to atherosclerosis via vascular inflammation [6], [7]. Herein, we have clearly shown that the mRNA levels of *ICAM-1, VCAM-1* and *MCP-1* were upregulated by Ang II, although their expression was remarkably reduced to normal levels in animals treated with β-carotene and α-tocopherol. Therefore, current investigation is in line with a previous report showing that all the genes expressed herein could be due to the role of β-carotene in downregulating inflammatory genes implicated in the development of AAA [34].

Together, these data suggest that dietary supplementation of β-carotene protects against Ang II-induced AAA by ameliorating macrophage recruitment in *Apoe^−/−^* mice. Our study is also suggestive of the role of certain inflammatory genes for instance, *VCAM-1, MCP-1, MMP-2, MMP-9, MMP-12, PPAR-γ* and *uPAR* in the pathogenesis of AAA. The pre- and post-treatment gene expression profiling have yielded some basic insights into use of α-tocopherol and β-carotene in ameliorating these inflammatory genes in Ang II-induced AAA. The results clearly indicate that β-carotene may activate *RAR/RXR* pathway leading to dimerization with *PPAR*s to repress the target genes, especially *MMP-2, MMP-9* and *MMP-12* to regulate inflammatory reactions in AAA in *Apoe^−/−^* mice.

## Materials and Methods

### Animals and Materials

Experiments were conducted according to the guidelines formulated for care and use of animals in scientific research (Indian National Science Academy, New Delhi, India) at a CPCSEA (Committee for the Purpose of Control and Supervision of Experiments on Animals) registered animal facility. The experimental protocols were approved by the Institutional Animal Ethical Committee (IAEC) at CCMB (agreement no. IAEC72/07). We used 4-months-old male apolipoprotein E (*Apoe^−/−^*) knockout (*n = 36*) and control mice in our experiments, purchased from The Jackson Laboratory, ME, USA. Ang II was procured from Sigma-Aldrich, and ALZET osmotic pumps were from Charles River Laboratories.

### The Mouse Model of Angiotensin II–Induced Aortic Aneurysm

Aneurysm was induced by Ang II– infusion as described previously by others in *Apoe^−/−^* mouse models [18]. Briefly, the mice were subcutaneously administered with Ang II via ALZET osmotic pump implantation [1], [16], [18], at a dose of 1.44 mg/kg/day for 45 days. *Apoe^−/−^* control mice (*n = 6*) received normal saline. After 45 days, 6 treated and 6 control *Apoe^−/−^* mice were sacrificed as per standard euthanastic protocols. The remaining 24 Ang II-treated *Apoe^−/−^* mice were grouped into four groups with six animals in each group. The first group received 800 mg α-tocopherol/kg of feed; the second received 800 mg β-carotene/kg of feed, the third group received 800 mg α-tocopherol and 800 mg β-carotene/kg of feed and the fourth group received normal chow diet. The antioxidant was mixed with normal chow diet ingredients and formed into pellets (National Institute of Nutrition, Hyderabad). After 60 days, antioxidant-treated and control *Apoe^−/−^* homozygous mice were sacrificed. The size of abdominal aorta was measured in control saline-treated, Ang II-treated and different antioxidant-treated *Apoe^−/−^* mice. The abdominal aortic size was measured at the time of sacrifice in the control saline-treated, Ang II-treated and antioxidant-treated *Apoe^−/−^* mice using vernier caliper.

### Biochemical Analysis

Prior to euthanasia, 0.5 ml of blood was collected from each mouse by orbital sinus puncture, and serum was separated by centrifugation at 1500rpm for 15 minutes. Serum total cholesterol (TC), high-density lipoprotein (HDL), low-density lipoprotein (LDL) and triglyceride (TG) were measured by enzymatic methods using spectrometry (Crest Biosystems, Pune, India).

### Histopathological Analysis

Complete gross and histopathological evaluations were carried out in control and antioxidant-treated *Apoe^−/−^* mice. After euthanasia, aorta and viscera such as heart, liver, spleen, kidney and brain were excised and dispensed in 10% buffered formalin (Sigma) from the control and treated *Apoe^−/−^* mice. Fixed and paraffin embedded tissues were cut at 5 µm thickness, stained with haematoxylin and eosin and examined under a light microscope (Zeiss, Germany).

### Flow Cytometry

Peripheral WBCs were isolated by gravity sedimentation of whole blood on 3% dextran in Hank’s balanced salt solution followed by red cell lysis in hypotonic shock. The WBCs were resuspended in fluorescence-activated cell sorting (FACS) staining medium (phenol red-free Hank’s balanced salt solution supplemented with 6% fetal bovine serum (FBS) and 0.01 mol/L Na^2^-EDTA). Subsequently, WBCs were stained for 1hr at 4°C in the dark with fluorescein isothiocyanate (FITC)-labeled anti-mouse CD45.2 and FITC-labeled anti-mouse MAC3 (1∶100) separately for each sample followed by washing and resuspending in FACS staining medium. Later, the samples were acquired on a BD FACSCalibur (Becton Dickinson, GmbH, Germany). Results were analyzed using FlowJo analysis software (Tree Star).

### Confocal Microscopy

The frozen sections (15 µm) of treated and control aorta were fixed with acetone for 20 minutes followed by permeabilization with 0.5% (v/v) Triton X-100 for 10 minutes at room temperature. Following blockade with 5% fetal calf serum (FCS) for 1 hour, the sections were incubated with mouse monoclonal antibody (Mac3 (CD107b), CD45.2, CD54 (ICAM-1), vascular endothelial growth factor (VEGF) and retinoic acid receptor (RAR) for 1hr at room temperature. The sections were subsequently washed thrice with 1% PBS and incubated with anti-mouse secondary antibody conjugated with FITC for 1 hour. To reduce autofluorescence, the sections were treated with CuSO_4_ (10 mM) in ammonium acetate buffer (50 mM, pH5.5) for 30 minutes. The sections were counterstained with propidium iodide (PI) for 5 minutes and mounted in vector shield (Vector Laboratories, CA, USA). Confocal laser scanning immunofluorescence microscopy (CLSM) was carried out using a Zeiss LSM 510 META confocal microscope. Image analysis was done using LSM510 META software (Carl Zeiss, USA).

### cDNA Synthesis and Quantitative Real-Time PCR Amplification

Quantitative real-time PCR (qRT-PCR) was performed on an ABI Prism 7700 (Applied Biosystems) in triplicate. Gene expression of adhesion molecules, cytokines, MMPs, plasminogen activator system (PAS), peroxisome proliferator-activator receptors, RARs and retinoic X receptor (RXRs) systems was determined by qRT-PCR. To quantify the relative levels of mRNA message, aortic tissue samples of different groups, including *Apoe^−/−^* mice treated with Ang II, α-tocopherol, β-carotene, α-tocopherol and β-carotene cocktail and control group were homogenized and total RNA was extracted with TRIzol® reagent (Invitrogen, USA). Subsequently, total RNA was measured by spectrophotometry (NanoDrop1000, DE, USA) at 260 nm. Purity of RNA was assessed by the quotient of 260/280 nm and integrity was confirmed by agarose gel analysis. Each sample was normalized to 1 µg of total RNA, and cDNA synthesis was performed using the first-strand cDNA synthesis kit (SuperScript® III Reverse Transcriptase, Invitrogen, USA) primed with oligo(dT)20 in a 50 µL reverse-transcription reaction on a GeneAmp 2400 thermal cycler system. The reverse-transcription products (cDNA) served as the template for qRT-PCR analysis, with gene-specific primers, reagents and protocols provided in the SYBR® Green PCR kit. All gene-specific primers were selected using GeneTools software to amplify a 150–200bp product with a T_m_ of 55–60°C. The primers used are shown in **File S3**.

### Statistical Analysis

Values are expressed as percentage or mean ± SE when appropriate. Comparisons and correlations of treated and control samples were made with paired t test. Statistical analysis was performed with GraphPad Prism 5 software (GraphPad Software, Inc., La Jolla, CA, USA). P values of less than 0.05 were considered significant.
